# Head Injuries in Wartime

**Published:** 1940

**Authors:** F. Wilfred Willway

**Affiliations:** Consultant in Head Injuries, S.W. Section, Ministry of Health; Surgeon, Burden Neurological Institute; Asst. Surgeon, West End Hospital for Nervous Diseases


### HEAD INJURIES IN WARTIME

BY

F. Wilfred Willway, M.D., M.S., F.R.C.S.,

*Consultant in Head Injuries, S.W. Section, Ministry of Health ;*
*Surgeon, Burden Neurological Institute ;*
*Asst. Surgeon, West End Hospital for Nervous Diseases.*

---

Regarding the incidence of head injuries in modern warfare, it is obvious that figures taken from the records of the Great War cannot be applied to present conditions. It is perhaps reasonable to assume that in air bombing some 10 out of every 100 injuries will be head injuries. Of these maybe three will be severe untreatable cases ; four, closed injuries (with no open wound), and three will have open wounds of the skull, scalp and brain. It will be noticed that the incidence of closed injuries is placed higher than open injuries ; this may be surprising, but is probably true and will be alluded to later. Thus a large raid with say 5,000 casualties will provide 350 head injuries requiring treatment. The purpose of this article is to describe the form these injuries may take.

### Classification.

Head injuries may be grouped into major categories as follows :—

A. Open wounds of the head.
  1. Scalp wounds pure and simple.
  2. Scalp wounds with symptoms of intracranial lesions.
  3. Open fractures of the skull.

B. Closed head injuries.
  1. Simple concussion cases.
  2. Cerebral irritation cases.
  3. Intracranial hæmorrhage cases.

C. Late " problem " cases.

It is not claimed that this is an exhaustive list or even a very scientific method of classifying such cases. But it supplies a rough and ready means of grouping cases in the hospital admission department.

It is clear that many patients will have multiple injuries : in some cases the head injury being the major feature, in others the head injury being mild and the other injuries dominating. The groups listed above will now be considered in more detail.

### A. Open Wounds of the Head.

### 1. *Simple Scalp Wounds.*

Providing there has been no loss of consciousness, these cases will not ordinarily require detention. Momentary stunning will probably not be considered as loss of consciousness sufficient to warrant detention in view of heavy pressure upon available beds. The larger first-aid posts and the minor surgical teams will deal with these cases by excision and suture. Free hair cutting and wide shaving is essential. Three special features may be mentioned, namely : severe hæmorrhage, difficulties in closure, and major loss of scalp-tissue. If no fracture exists hæmorrhage may be temporarily controlled by a scalp tourniquet and by under-running sutures. Permanent control is secured by accurate re-suturing in two layers, Difficulty in approximation can be overcome by either an S-shaped or tripod extension of the wound ; hence the importance of the preliminary wide shaving. Extensive loss of scalp substance must cause difficulty. Such wounds after debridement are best treated by immediate Thiersch grafts, which, when bandaged on firmly, take extremely well. Local anæsthesia will suffice for all cases in this class. Dressings are difficult to apply and uncomfortable to wear in these cases. The wounds should be painted with mastisol or Whitehead's varnish and then left to dry, except skin-graft cases, where the special dressing of tulle-gras, etc., should be used.

### 2. *Scalp Wounds with Symptoms of Intracranial Lesions.*

This is a common type of civil injury and it is also likely to be common in warfare. In the majority of cases the intracranial symptoms will take precedence and once the scalp wound has been dealt with, this group will then fall into the category of closed head injury (p. 94). Any severe head injury causes a good deal of collapse, which may last some hours. During this period it is most inadvisable to deal with wounds even though contamination is present (all " raid " cases may be considered to be contaminated). A nice dilemma arises between the natural desire to excise contaminated tissue at the earliest possible moment and the need to leave shocked cases alone. Local anæsthesia makes it possible to excise at an earlier moment than would otherwise be possible Some American surgeons press the policy of delay, and say it does not lead to infection if the wounds, once covered, are then left undisturbed.

The problem will arise in the cases in this group and the next.

3. *Open Skull Fractures.**

These will be present in four groups :—

(*a*) Head injuries with scalp wounds where exploration shows a fissured fracture lying in the depths of the wound.

(*b*) Head injuries where a compound fracture is immediately obvious but the dura mater is apparently intact.

(*c*) Gross compound fractures with protusion of brain substance.

(*d*) Fractures of the base of the skull which have to be catalogued as open fractures (except the rare posterior fossa fracture).

A fracture of the skull is of paramount importance when it is :—

Compound : Leading to infection of the brain and meninges.
Depressed : Compressing the brain.
A cause of lacerated blood vessels : leading to compression of the brain.
Associated with injury to cranial nerves.

Fracture of the skull may be of diagnostic significance, indicating the site of cerebral damage. Fracture of the skull may occasionally provide a spontaneous decompression. Apart from the above provisos a skull fracture is a matter of surgical insignificance, though frequently of legal importance.

It will be noted that from the foregoing that fracture is only important in so far as it causes cerebral damage.†

Certain features of the four categories listed will now be mentioned :—

(*a*) The routine treatment for the scalp wound is undertaken, with the necessary measures for the concomitant cerebral damage. No special treatment is necessary for the fracture unless obviously depressed, or unless focal symptoms or compression symptoms are present.

It is not denied that in some few of these cases there may be a separation or depression of the inner table of the skull only, but in the absence of symptoms immediate interference is undesirable. It is preferable to wait for detailed radiological studies, the electro-encephalogram and other special tests.

(*b*) The group of head injuries where a compound fracture is immediately obvious, but the dura mater is apparently intact,

* It is at present recommended to give sulpha-pyridine to all open head injury cases *before* the occurrence of symptoms suggestive of infection. As soon as the patient can swallow, tablets of M. and B. 693 are given. Its benefit in certain cases is undoubted.

† It was said of these fractures at the time when the "Bohler treatment" of general fractures received world publicity, that skull fractures should be treated by a Bowler hat. This witty remark, I believe, is attributed correctly to Sir William De Courcy Wheeler. It is appropriate, in that emphasis is placed on the wisdom of "no interference."

frequently provides gratifying results. The force of the blow appears to have expended itself in damage to the brain-case, while the precious contents may sometimes be singularly little damaged. The principles of wound excision are meticulously applied, the scalp edges being first excised and then the bone edges trimmed away. Comminuted fragments are removed, and it is of no moment if wide skull defects are left. Bleeding is arrested and the dura mater inspected.

Should the dura mater be opened? There are definite indications, one definite contra-indication and some doubtful cases. The indications are neurological symptoms of focal cerebral damage, general symptoms of compression—stupor (many patients in this class will be conscious on admission)—or bluish coloration of the dura indicating sub-jacent hæmorrhage. The contra-indication is established infection in late cases.

At the inspection of the dura mater small unsuspected lacerations may be found; these are sutured if there is no tension.

(*c*) Gross Fractures.—These are not necessarily fatal, though the prognosis is certainly much worse. Perhaps 50 per cent. may be saved, though not all of these will return as economic members of society. After excision of the scalp edges and free removal of bone, pulped brain tissue should be gently removed by irrigation and suction. The dural edges must not be excised nor retracted, as this is liable to open up the leptomeningeal spaces and to allow a local infection to become generalized. The wound-track in the brain must be gently explored and cleared of débris. Before closure the cortex will be protected by a sheet of amnio-plastin. Drainage of such wounds is a wise precaution.

(*d*) Fractures of the Base of the Skull.—Here the word "management" is preferable to "treatment" as indicating that there is not necessarily much to do but plenty of scope for careful supervision. The nasal passages and the external auditory meatus must not be plugged because of hæmorrhage or leakage of cerebrospinal fluid: nor should these passages be irrigated or syringed. The external meatus should be gently cleansed with cotton wool mops soaked in spirit by someone who can use a speculum. A wick of gauze in carbolic 1 in 80 may be left in the meatus and a sterile covering dressing applied. Treatment otherwise will be directed to the neurological state, and this is considered under the following sections.

### B. CLOSED HEAD INJURIES.

The importance of making notes at the earliest opportunity should be emphasized. Notes made by first aid parties *at the time of observation* giving *date* and *time* as well as clinical facts are very valuable. Particular attention should be paid to the state of consciousness, *i.e.* normal consciousness, conscious but confused, irritable, semi-conscious, some reflexes, unconscious, reflexes lost and deep coma. The cranial nerves can be quickly tested and the condition of the limbs, whether paralysed or normal, and the state of limb reflexes noted.

Intra-cranial hæmorrhage, which may be remediable, is usually diagnosed by observing a "march of events" in the clinical picture; it is the recorded changes which cause the surgeon to suspect a hæmorrhage: hence the importance of accurate note-taking.

*Simple Concussion Cases.*

These cases call for observation rather than treatment. The diagnosis is confirmed in retrospect rather than at the time of examination. It is not possible to be certain that an intracranial hæmorrhage has not occurred until the patient has remained well over a period of time which may have to be months. Of course, it is always possible to make a shrewd guess sooner and it is certain that the first forty-eight hours are the most critical.

On the other hand, experienced neuro-surgeons can recall cases apparently well and discharged from hospital who have been re-admitted on account of hæmatomata. The writer has a case in mind :—

M.C. (a sailor, age 23 years) had been two weeks in hospital for observation after "severe concussion"—he was apparently well and the attendants were not concerned about him. He was evacuated in the "Munich crisis," but re-admitted some fourteen days later in coma. I then saw him for the first time, diagnosed and operated on a huge sub-dural hæmatoma. Unfortunately, he never regained consciousness, and four days after operation he died. In my opinion cortical asphyxia had lasted too long before operation to permit recovery.

Most concussion cases make a rapid and complete recovery. If this does not occur, suspicion is aroused that the case is not one of concussion at all, and some other diagnosis must be sought for. The immediate treatment of concussion is rest and quiet.

Since most cases are almost well next day, three weeks in a darkened room is an intolerable imposition. While prolonged rest is advisable, convalescence must be judiciously managed: books, jigsaw puzzles and wireless allowed if the patient wants them: they may dispel boredom and introspection. Many patients will refuse to rest at all and may suffer no penalty for refusing advice which is clearly only dictated by caution. Perhaps "living dangerously" should not be discouraged at the present time!

A few will develop the post-concussional syndrome (headaches, inattention, anxiety feelings, minor forgetfulness, irritability) and require expert investigation (electro—and/or air—encephalography), or the aid of a psychologist.

*The Cerebral Irritation Case.*

This is a familiar and not uncommon clinical type. The patient lies curled up, resenting light, any examination, or nursing attention. He is not unconscious and can easily be aroused and sometimes irritated to frenzy. He may be restless, and then a search should be made for non-cerebral irritants. A common example is a full bladder. The bemused patient may keep trying to get out of bed to micturate while the nurse keeps pushing him back, until a fight may develop.

An external irritant will not always be found, but it should always be looked for. Complications in cases of cerebral irritation include intracranial hæmorrhage, bronchopneumonia, urinary infection and bedsores.

*Should Dehydration Therapy be Employed?*

A few years ago this was a popular routine : at the present time its *routine* use is condemned. For selected cases and for specific purposes (*e.g.* cases of general cerebral oedema, to relieve coma for pre-operative diagnosis, to relieve tension temporarily until decompression is possible) it is definitely valuable. Dehydration methods may be classified as slow and rapid. Standard *slow* methods are : magnesium sulphate in large doses by mouth or 6 to 10 oz. of common salt 20 per cent. solution per rectum, six hourly or p.r.n. If persisted with, symptoms of proctitis may supervene, and the treatment must be stopped.

*Rapid* dehydration is obtained by either intravenous injections, 30 per cent. sucrose in doses of 50 to 200 c.cm, or by lumbar puncture. Diagnostic lumbar puncture with manometry must precede and control rapid dehydration measures.

Dehydration cannot be beneficial and must be harmful if there is intracranial bleeding or damage to cerebral substance : dehydration cannot be harmful and will be beneficial if generalized cerebral oedema is the sole lesion that exists. Thus accurate diagnosis must precede this treatment.

Most cases of cerebral irritation, if the major complications, as previously described, are avoided, will gradually improve and return to normal, or almost normal, in from three weeks to six months. Some cases, however, do not do so : the problem then is to decide whether operative measures should be employed. Exploration is justified if there is any likelihood of a localized hæmorrhage, or even if doubt exists in a case where normal improvement is not taking place. In a case where manometry confirms the diagnosis of persistent oedema suggested by headache, irritability, papilloedema, and where control by dehydration is difficult, then a right-sided subtemporal decompression is justified.

*Intracranial Hæmorrhage Cases.*

These form the dramatic cases in head injury work. They are comparable to the ruptured spleens and ruptured ectopic gestations of general surgery, in that prompt and fearless diagnosis is necessary together with effective operative intervention. The comparison holds further in that it is probably safer (in good hands) to operate unnecessarily than to fail to operate in doubtful cases.

Intracranial hæmorrhage may be :—

1. Extra-dural.
2. Sub-dural : Diffuse, when it is also sub-arachnoid ; Localized.
3. Intracerebral : Small, leading to cyst formation ; Large, generally fatal.

Extra-dural hæmorrhage may occur from the middle meningeal vessels or from the superior longitudinal sinus or from the lateral sinuses.

Sub-dural hæmorrhage of the local variety may appear in an acute or chronic form, *i.e.*, symptoms may appear within a few days of the accident, or not until months or years after the accident, when a cerebral tumour may be simulated.

Hæmorrhage in the cranial cavity is usually of a progressive nature, and the diagnosis of its presence and situation is more important than the diagnosis of its type, whether extra or sub-dural. The early symptoms are irritative and thus difficult to distinguish from cases in Group B.2. Suspicion of intracranial bleeding is aroused by noting :—

1. Progressive symptoms.
2. Occurrence of convulsions.
3. Onset of paralysis.
4. Persisting unconsciousness.
5. Pupillary changes.
6. Persistent vomiting.

The site of the hæmorrhage is suggested by the presence of scalp bruising or skull fracture (this may be misleading), or by focal symptoms and mode of onset of the paralysis or other symptoms.

Success in diagnosis and subsequent treatment is often due simply to the awareness on the part of the medical attendant of the possibility of hæmorrhage. Too often in the past have cases of this nature been allowed to lie in bed to all intents and purposes unexamined, and left to live or die as nature wills. Knowledge of the pathological processes at work will often enable the surgeon to turn the scales in favour of recovery.

### C. LATE OR PROBLEM CASES.

This is a wide category comprising such apparently unrelated cases as :—

1. Late epilepsy.
2. Cerebral abscess.
3. Retained foreign body.
4. Persistent headache.
5. Cerebral hernia.
6. Skull defects, etc.

It is impossible in this article to deal with the diagnostic and operative features of these cases. They will provide the neuro-surgeon with his " interesting cases " and tax his ingenuity and skill.

### *Points in First Aid.*

1. Note-taking.—Brief, accurate notes from the first-aid posts are indispensable. So many head injury cases will be unable to tell their story that a brief note is invaluable. *E.g.* :—

(*a*) Hit by fallen roof tile, unconscious till arrival at Aid Post. Conscious but confused here. No paralysis noted.—X.Y.Z., Aid Post M.O., 4.0 p.m., 0/0/40.

(*b*) Brought in in irritable state. Jumped out of second floor escaping fire. Pupils equal and react. Corneal reflex brisk, ? paralysis left arm.—X.Y.Z., 1.0 a.m., 0/0/40.

2. Treatment.—Simply cover scalp wounds unless free hæmorrhage is still taking place. Treat bleeding cases by tourniquet if skull is intact, by suture if skull is fractured. **Do not** give morphia.

3. Transport.—Shocked and irritable cases must lie flat. Other cases may sit up.

